# Strangulated *Bochdalek* Hernia in an Adult Patient

**DOI:** 10.1002/ccr3.71536

**Published:** 2025-12-02

**Authors:** Maria Ana Mirante, Marco Serôdio, Lucília Conceição, Sílvia Borges, António Ribeiro Mendes

**Affiliations:** ^1^ ULS Médio Tejo Tomar Portugal; ^2^ ULS Coimbra Tomar Portugal

**Keywords:** bochdalek hernia, diaphragmatic hernia, emergency surgery, intestinal strangulation

## Abstract

Strangulated *Bochdalek* hernia is a rare, life‐threatening condition in adults that requires a high degree of suspicion for diagnosis. Symptoms are often nonspecific, and prompt imaging is crucial. Early surgical intervention is essential to prevent irreversible damage and reduce mortality.

## 1 Case Report

**FIGURE 1:**
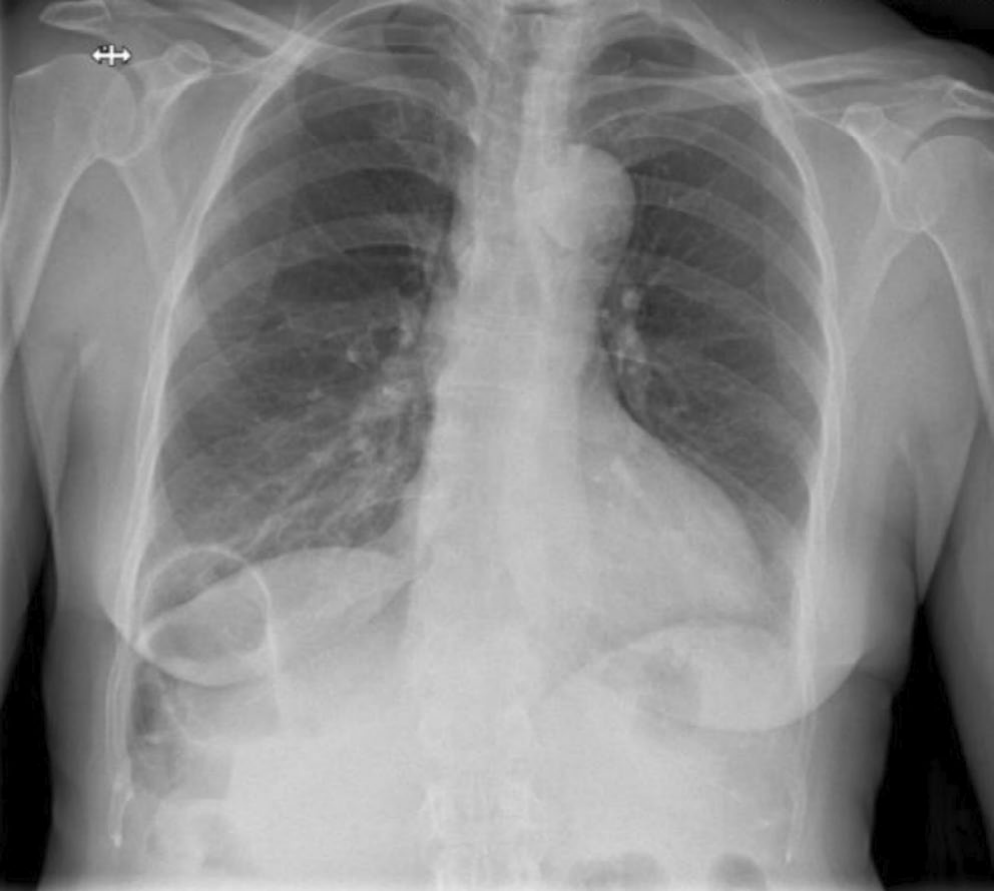
Chest x‐ray showing a bowel loop in the thoracic cavity.

A 65‐year‐old woman with a history of severe obstructive sleep apnea and 4 months of intermittent epigastric pain presented to the emergency department with sudden, unbearable epigastric pain and vomiting. She denied recent trauma. A previous chest x‐ray (Figure 1) had already shown bowel loops within the thoracic cavity.

**FIGURE 2:**
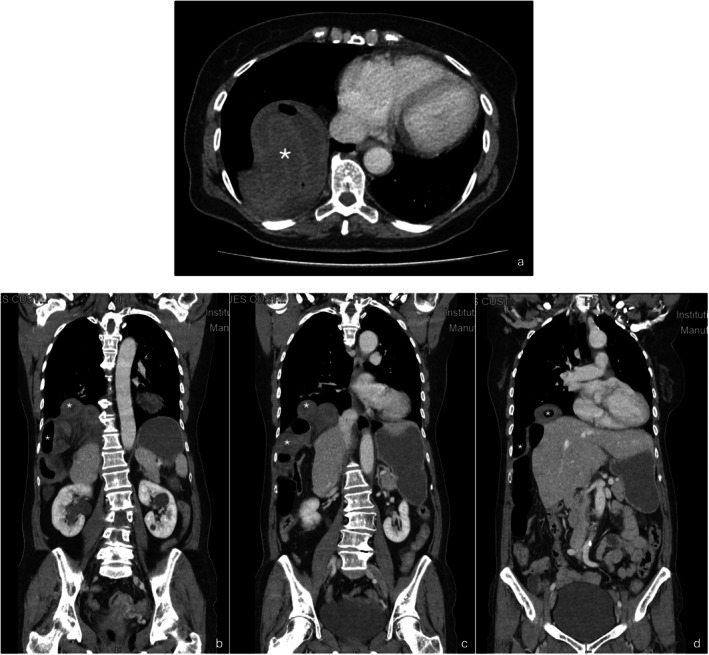
CT scan—transverse (a) and sagittal views (b, c, d) showing the bowel segments passing laterally to the liver and into the thoracic cavity (marked as *).

Laboratory tests revealed mildly elevated inflammatory markers, arterial blood gas with partially compensated metabolic alkalosis, and an elevated serum lactate level (6.3 mmol/L), suggesting tissue hypoperfusion. Electrocardiogram and troponin levels were normal, excluding cardiac etiology. CT confirmed a Bochdalek hernia with herniated small bowel loops showing signs of ischemia (Figure 2).

**FIGURE 3:**
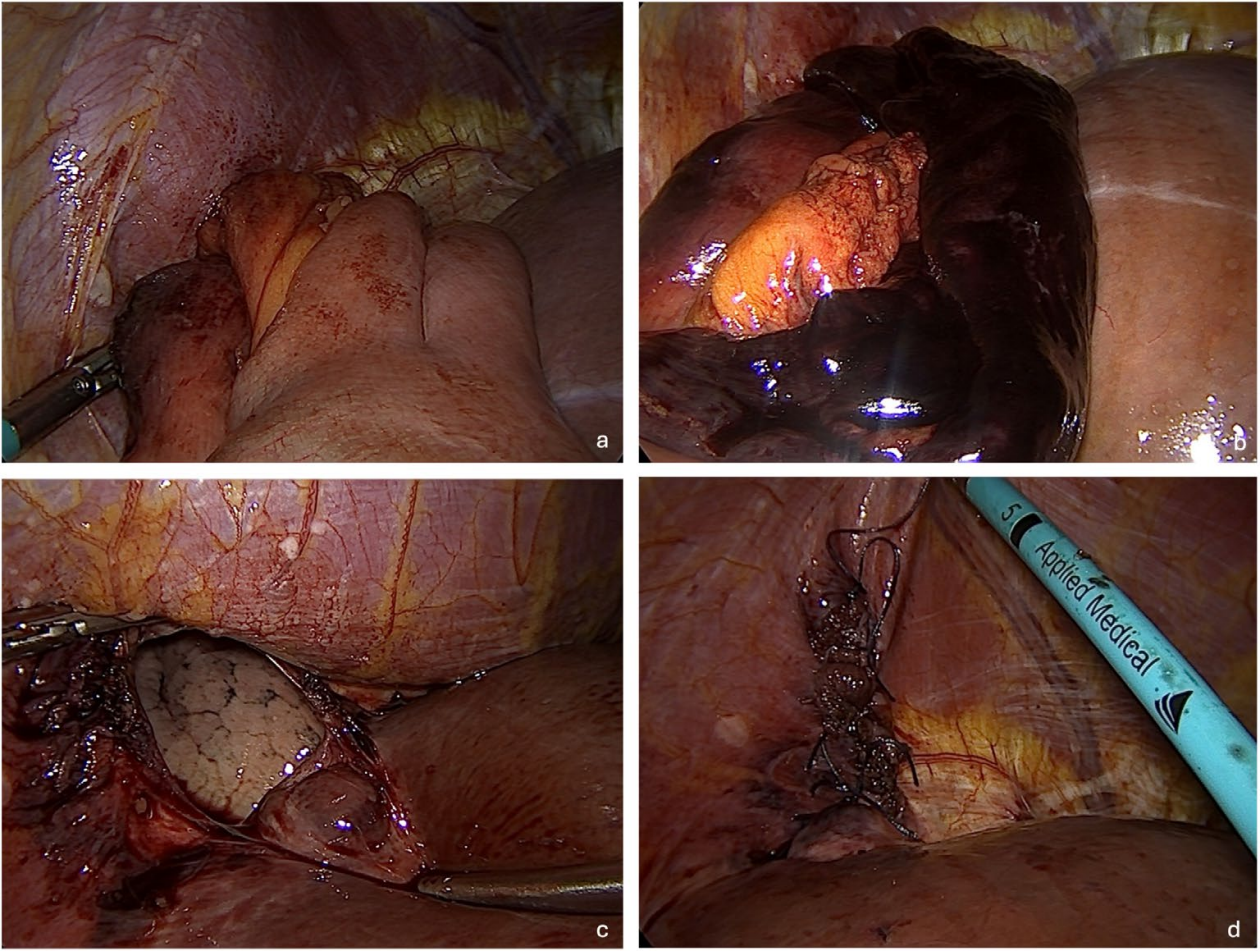
Exploratory laparoscopy images with evidence of bowel segments passing through the liver into the thoracic cavity (a); the ischemic bowel segment after partial reduction (b); the orifice in the diaphragm (c); closed diaphragmatic orifice after suture (d).

**FIGURE 4:**
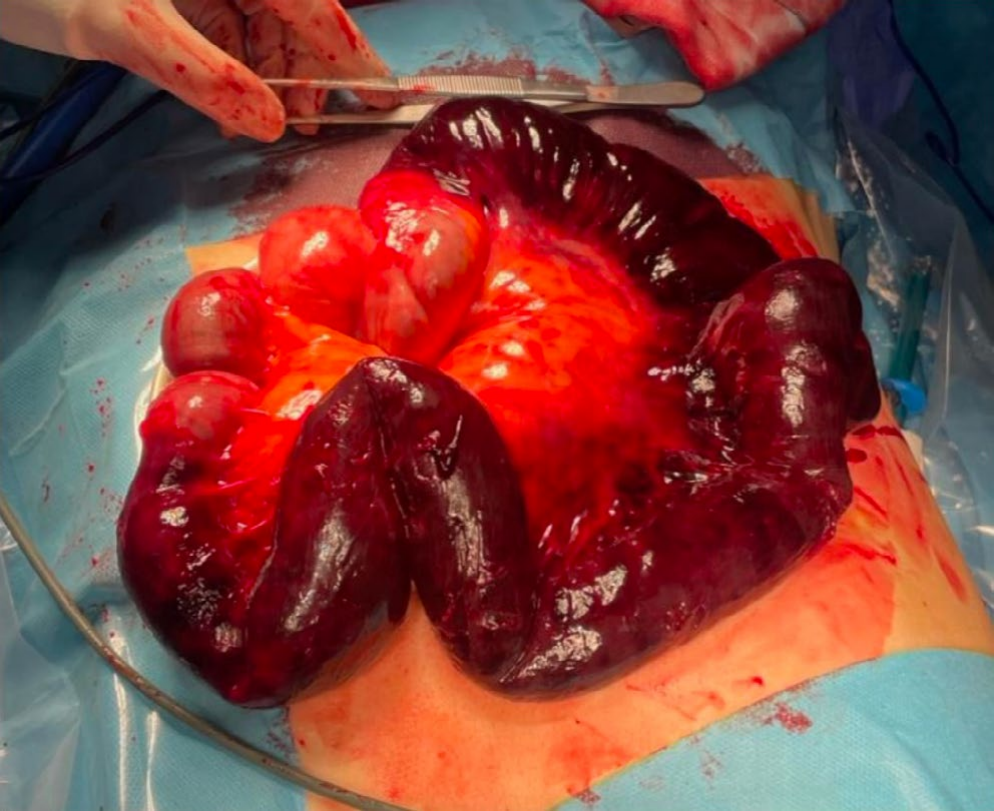
The herniated small bowel segment, with signs of ischemia.

Intraoperatively, approximately 50 cm of strangulated ileum was found within the thoracic cavity with irreversible necrosis. The herniated contents were reduced, the diaphragmatic defect was closed with sutures, and the necrotic bowel segment was resected (Figures 3 and 4). The patient was admitted to the intensive care unit for postoperative monitoring. Seventeen days after the surgical procedure, the patient was discharged from the hospital, completely recovered and asymptomatic.

**FIGURE 5:**
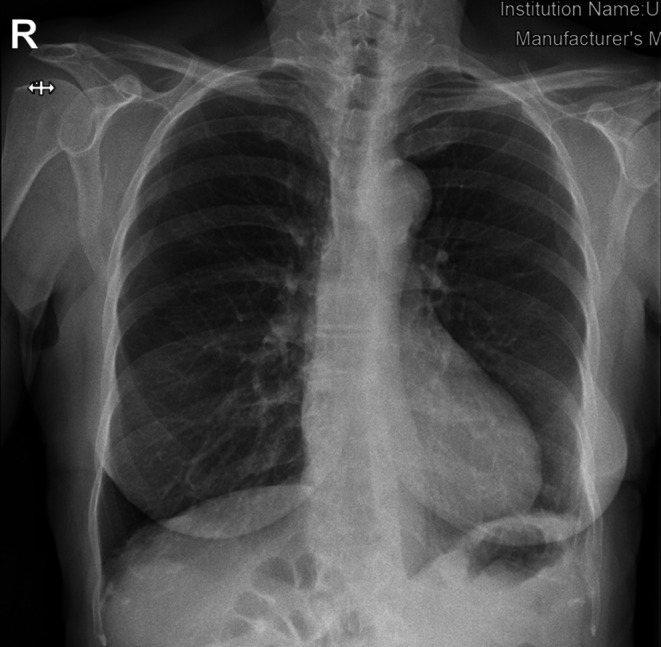
Four‐month follow‐up thoracic x‐ray.

At 5 months, there was no evidence of hernia recurrence (Figure 5) or respiratory complaints, confirming a favorable outcome.

Strangulated Bochdalek hernia in adults, although rare, is a life‐threatening emergency. The nonspecific nature of its symptoms requires a high index of suspicion, particularly in patients with a known diaphragmatic defect or incidental radiological findings [1]. Prognosis depends largely on the promptness of diagnosis and surgical intervention [2]. Early surgical repair is typically associated with excellent outcomes, while delays in treatment may lead to sepsis, multi‐organ failure, and high mortality [3]. Long‐term follow‐up is crucial to monitor for recurrence and any residual pulmonary or gastrointestinal dysfunction.

## Author Contributions

**Maria Ana Mirante:** conceptualization, data curation, formal analysis, writing – original draft. **Marco Serôdio:** formal analysis, supervision, validation, visualization, writing – review and editing. **Lucília Conceição:** supervision, validation, writing – review and editing. **Sílvia Borges:** supervision, validation, visualization, writing – review and editing. **António Ribeiro Mendes:** supervision, validation, visualization.

## Funding

The authors have nothing to report.

## Consent

Written informed consent was obtained from the patient to publish this report in accordance with the journal's patient consent policy.

## Conflicts of Interest

The authors declare no conflicts of interest.

## Data Availability

The data that support the findings of this study are available from the corresponding author upon reasonable request.
